# Retinal Artery Occlusion in Fibromuscular Dysplasia: A Case Report

**DOI:** 10.7759/cureus.31462

**Published:** 2022-11-13

**Authors:** Yusuf Mehkri, Jordan Poe, Minaal Murshid, Jairo Hernandez, Prashant Singh, Vikash N Sinha, Hans Shuhaiber

**Affiliations:** 1 College of Medicine, University of Florida College of Medicine, Gainesville, USA; 2 Neurology, University of Florida College of Medicine, Gainesville, USA; 3 Radiology, University of Florida College of Medicine, Gainesville, USA

**Keywords:** cardio vascular disease, visual changes, neurology case report, branch retinal artery occlusion, fibromuscular dysplasia

## Abstract

Fibromuscular dysplasia (FMD) is a nonatherosclerotic, noninflammatory vasculopathy with no identifiable underlying cause. Clinical manifestations of the disease typically occur at the site of occurrence. Ocular manifestations of fibromuscular dysplasia are rare but can occur in the form of central or branched retinal artery occlusions, which can cause painless monocular vision loss. We present the case of a 71-year-old female patient with FMD presenting with worsening visual acuity due to suspected right branch retinal artery occlusion. Pathology and imaging findings were consistent with classic FMD, and given our initial concerns for this patient, the rare ocular manifestations of this disease are highlighted.

## Introduction

Fibromuscular dysplasia (FMD) is a non-atherosclerotic, non-inflammatory vascular disease that most commonly affects renal and internal carotid arteries, though it can affect arterial beds all throughout the body [1]. Arterial layers are divided into three sections: intima, media, and adventitia, and the classification of fibromuscular dysplasia is dependent on the arterial layer in which the lesions predominate [1], with medial dysplasia being the most prevalent [2]. Circumferential or eccentric depositions of collagen characterize the lesions observed with fibromuscular dysplasia [3]. Medial dysplasia in particular is associated with the classic “string of beads” appearance on imaging that distinguishes it from other forms [1]. Clinical manifestations of the disease typically occur at the site of involvement [3]. The most common cases of fibromuscular dysplasia are those related to renal artery involvement (60-75%), followed by cases involving cervicocranial arteries (25-30%), visceral arteries (9%), and arteries located in extremities (5%) [4]. Retinal manifestations such as retinal artery occlusions are very rare and have been reported in only six cases in the literature [5].

A variety of genetic, mechanical, and hormonal factors have been associated with fibromuscular dysplasia, however, the cause of the disease is still unknown [1]. It most commonly affects women, representing 90% of all cases, with the mean age of onset being 52 years [6]. Cigarette smoking, hypertension, stress, and coagulation disorders have all been associated with an increased risk of developing FMD [1,3]. Genetic predisposition may also play a part, as there have been increased instances of the condition in patients with first-degree relatives affected by FMD [7]. A link between a family history of cardiovascular disease and fibromuscular dysplasia has also been identified [8]. While hormonal irregularities are believed to influence the onset of fibromuscular dysplasia [3], currently no association has been found between fibromuscular dysplasia and abnormalities of endogenous sex hormones or the use of oral contraceptives [8].

## Case presentation

This is a case of a 71-year-old female with a history of hypertension, Parkinson’s disease, and recent deep brain stimulation (DBS, status post removal one month prior due to concern for infection), who presented to the emergency department (ED) for evaluation of progressive right eye blurry vision and photophobia. Her symptoms began two weeks prior to admission when she noticed an acute onset area of grey blurry vision in the right lower quadrant of her right eye. Her ophthalmology evaluation three days later found visual acuity to be 20/40 PHNI (pinhole no improvement) OD (oculus dexter), 20/40 PH (pinhole) 20/25 OS (oculus sinister), and normal anterior exam. Fundoscopic exam revealed mild optic disc edema with splinter hemorrhage OD, disc at risk OS. Her most recent labs had shown an ESR (erythrocyte sedimentation rate) of 30 and a CRP (C reactive protein) of 7.75, but the patient had been receiving IV (intravenous) vancomycin for several weeks prior to the exam for her infected DBS removal surgery. Additionally, the patient endorsed experiencing occasional episodes of hypotension. The ophthalmology report concluded there was a high suspicion of non-arteritic anterior ischemic optic neuropathy (NAION), and she was prescribed dorzolamide.

Six days later, she experienced an acute sensation of a "dark curtain falling down on her right eye" and a widening of her gray border. She was evaluated by neuro-ophthalmology five days later. On examination, she was found to have worsening visual acuity to 20/70 eccentric OD with a missing nasal field OD on (confrontation visual field) CVF testing. Humphrey visual field (HVF) testing was uninterpretable due to high unreliability; the right eye appeared diffusely depressed, while non-specific constriction was noted for the left eye. Pupillary reflexes and extraocular movements were intact bilaterally. External and slit lamp exams were unchanged from baseline, but her fundoscopic exam now showed 360° edema and margin blurring OD with four splinter hemorrhages inferiorly, one temporally, and one nasally. Disc pallor and mild peripapillary atrophy OS were noted. Optical coherence tomography retinal nerve fiber layer (OCT RNFL) measurements revealed a swollen disc OD at 220 μm.

Given the rapid progression and severity of her symptoms, an ischemic cause for her optic disc edema became less likely. Her previous mild elevations in ESR/CRP values highlighted the possibility of underlying inflammation, and considering her recent surgical history, CNS infection could not be excluded. Thus, her neuro-ophthalmologist referred her to our ED for further workup.

Upon presentation, the patient was noted to be mildly disoriented. She endorsed photophobia and visual disturbances as previously detailed and denied any loss of consciousness, nausea/vomiting, headache, periorbital ecchymosis, or pain. On exam, her vital signs were normal and her ophthalmic findings were unchanged from her previous neuro-ophthalmology exam. Her physical exam was otherwise normal. Laboratory findings revealed normal values for ESR/CRP. 

Non-contrast computed tomography (CT) images of the head revealed mild prominence of the trigeminal cisterns with minimal tortuosity of the optic nerves and subtle flattening of the right optic nerve head. There was no evidence of hemorrhage. A subsequent lumbar puncture found an opening pressure of 10 and a CSF profile not concerning infection. Orbital MRI demonstrated flattening of the right optic nerve head. There were nonspecific findings of questionable asymmetric mild enhancement around the right intraorbital optic nerve and within the cavernous sinus, suggesting inflammatory changes. Neurology was consulted and a temporal artery biopsy was recommended for concern of possible giant cell arteritis. The patient was empirically given 1g IV solumedrol in the ED and admitted to inpatient.

**Figure 1:**
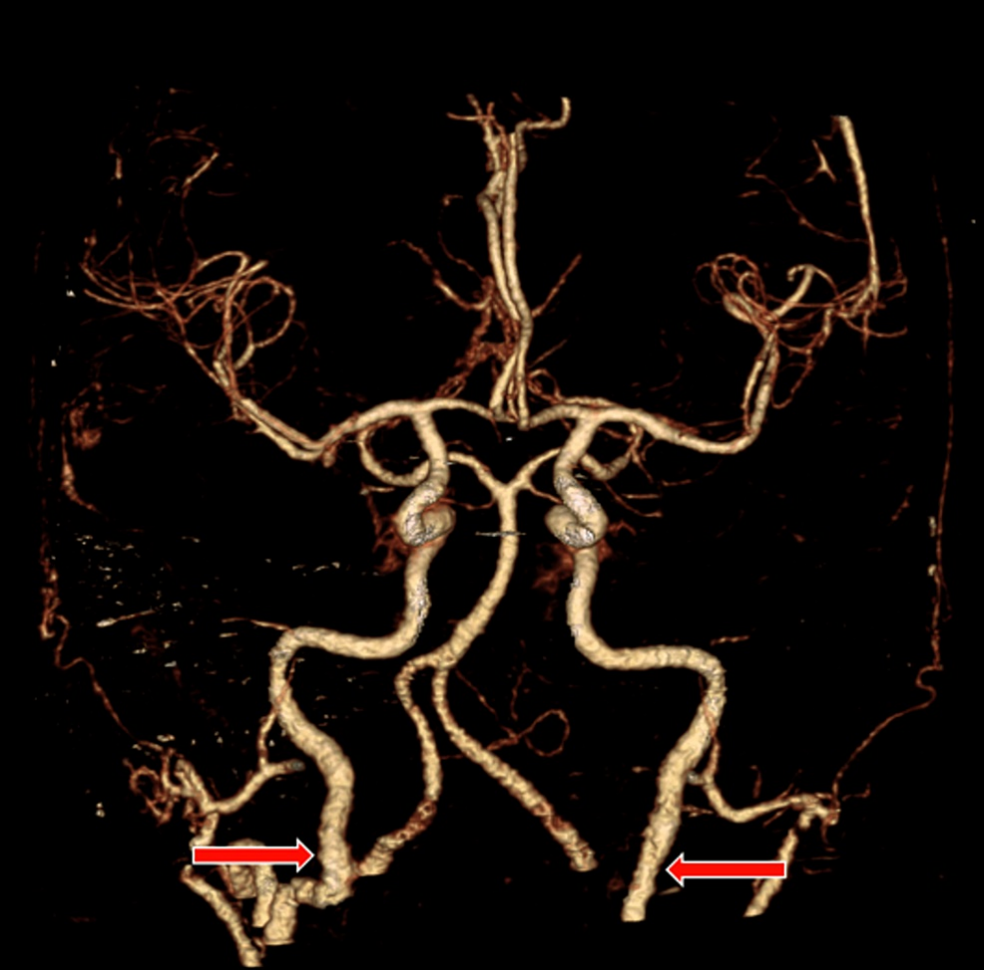
3D Volume rendered CT angiogram The angiogram demonstrates no intracranial arterial abnormality. Mild contour irregularities of both cervical internal carotid arteries are partially seen (red arrows).

**Figure 2:**
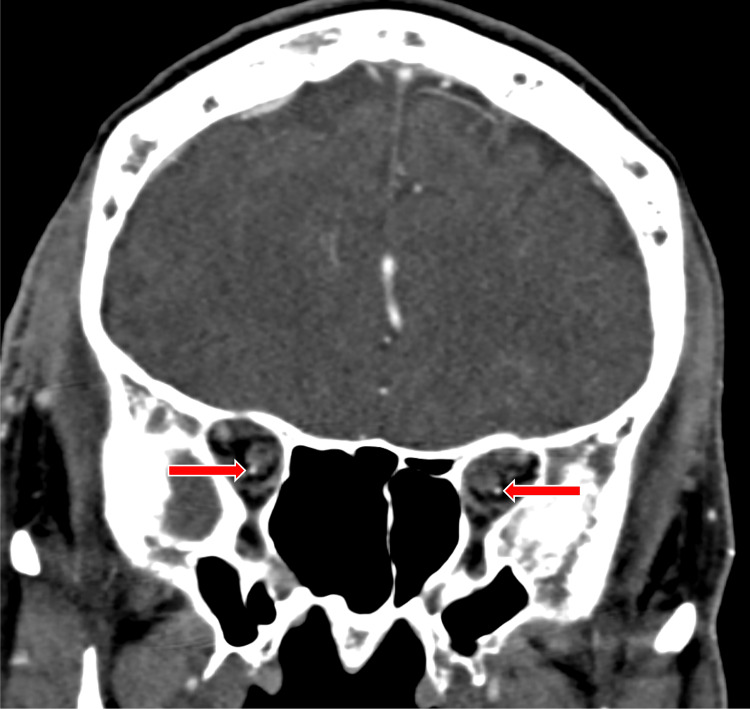
Coronal CTA image The computed tomography angiography (CTA) image demonstrates patent ophthalmic arteries bilaterally in the orbits (red arrows).

**Figure 3:**
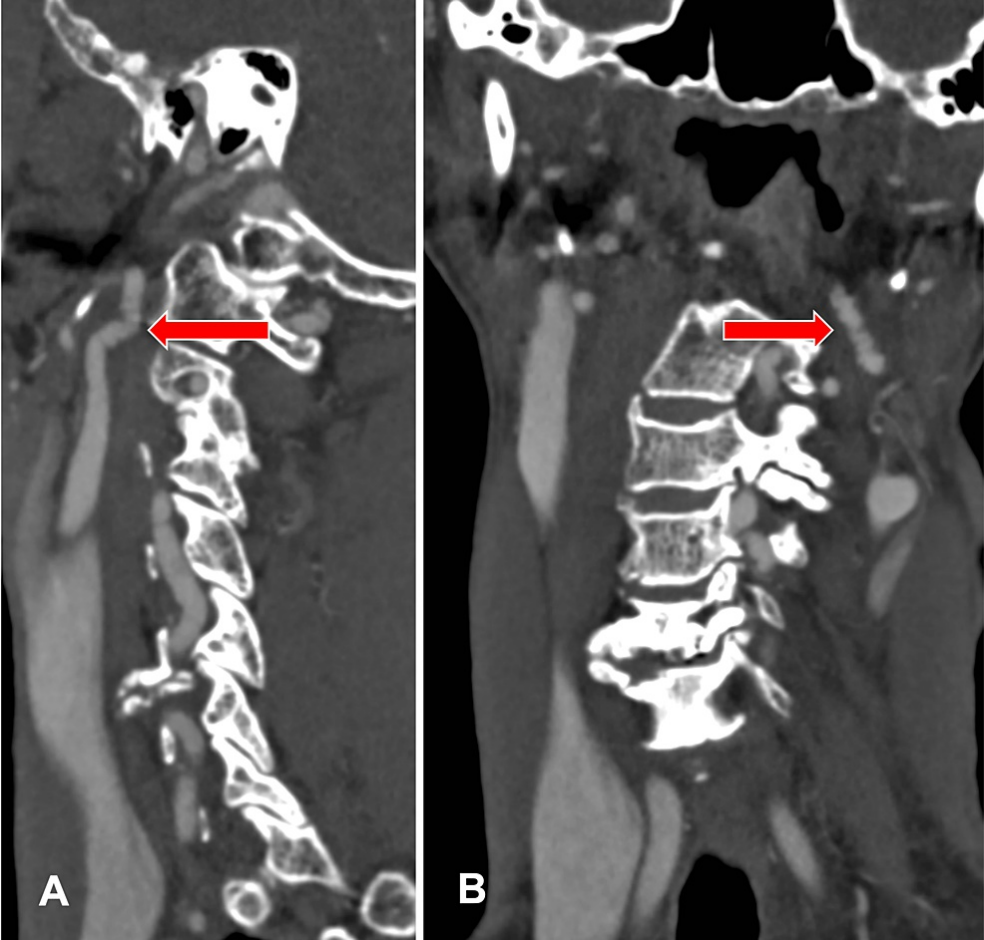
Sagittal CTA of the right internal carotid artery Sagittal computed tomography angiography (CTA) of the right internal carotid artery (A) and coronal CTA of the left internal carotid artery (B) shows alternating mild stenosis and dilatation, causing a string of beads appearance bilaterally (red arrow).

The biopsy was performed on day three of admission; the pathology report eventually found no evidence of acute vasculitis or inflammation. In the interim, the patient continued to receive solumedrol pending biopsy results. Imaging was obtained, and ophthalmology was consulted for a re-examination of her right optic disc edema. Ophthalmology found improved visual acuity to 20/40 PH 20/30 eccentric OD, and the rest of the exam was unchanged from the previous. An MR of the brain did not show strokes in a pattern that would explain her symptoms; chronic lacunar infarction in the left cerebellar hemisphere on a background of mild chronic microvascular ischemic changes was noted, but no acute abnormality or acute/subacute infarct. Computed tomography angiography (CTA) and MRI/MRA (magnetic resonance angiography) of the head and neck found no evidence of large vessel occlusion, flow-limiting stenosis, or aneurysm in the anterior or posterior intracranial circulation (Figure 1). Ophthalmic arteries were found to be patent bilaterally (Figure 2). Sagittal images demonstrated relatively symmetric focal luminal irregularity of the distal cervical internal carotid arteries bilaterally, giving a "beaded appearance" with similar, though less pronounced, luminal findings involving the distal cervical vertebral arteries bilaterally (Figure 3).

Imaging was most consistent with fibromuscular dysplasia and suspected right branch retinal artery occlusion (BRAO). Renal duplex studies showed patent renal veins bilaterally. The patient was discharged from the hospital with prescriptions for low-dose oral aspirin and high-dose slow-taper oral steroids. At her two-week post-discharge appointment, the visual acuity in her right eye had not improved, but her optic nerve OCT RNFL findings had improved to 86 μm OD. Given no clinical benefit from steroid therapy, she was successfully weaned off and a plan was made for a follow-up in three months to monitor progression and to continue aspirin therapy.

## Discussion

Fibromuscular dysplasia is a type of arteriopathy that can affect arterial beds all throughout the body and is considered a leading cause of systemic hypertension due to renal arterial stenosis secondary to renal artery involvement of fibromuscular dysplasia [3,6]. Fibromuscular dysplasia treatment varies based on severity, with stenting or balloon angioplasty in some cases, while asymptomatic individuals are managed medically and monitored on antiplatelet therapy [5].

Risk factors of fibromuscular dysplasia include smoking, hypertension, genetic, and hormonal factors. The association between smoking and fibromuscular dysplasia is largely undefined, however, there is a higher incidence of smoking among those diagnosed with fibromuscular dysplasia in comparison to matched hypertensive control subjects [5]. Smoking is already associated with an increased risk of aneurysms and further appears to increase the risk of aneurysms in patients with fibromuscular dysplasia [9], in which the risk for aneurysmal disease is already elevated [5]. Additionally, early diagnosis of fibromuscular dysplasia may have been delayed in patients who smoke since earlier symptoms could have been incorrectly attributed to smoking-related illnesses such as cardiopulmonary disease [9]. There appear to be multiple mechanisms by which smoking increases the risk of developing fibromuscular dysplasia [10]. These include endothelial dysfunction, promotion of a chronic, pro-inflammatory state, increase in insulin resistance, as well as an atherogenic lipid profile [10].

In cases of hypertension, arteries experience an increased blood flow pressure, adding excess force to arterial walls. A prolonged increase in blood pressure can damage vessel endothelium and lead to atherosclerosis [11]. In rarer cases, hypertension may lead to fibromuscular dysplasia as the abnormal collagen deposits on arterial layers may further narrow an already compromised vessel, which in turn would further worsen hypertension [12].

To date, no causative genes of fibromuscular dysplasia have been identified [6]. Through pedigree analysis, it has been concluded that the inheritance pattern is likely a dominant trait with reduced penetrance. In one study, 20 families with a history of fibromuscular dysplasia were examined, and it was found that 60% of cases of fibromuscular dysplasia were caused by an autosomal dominant trait with variable penetrance, while the remaining 40% were likely caused by de novo mutations [13]. However, in more recent studies confirmed with imaging, approximately 11% of fibromuscular dysplasia cases are classified as familial [6]. Genetic investigation of fibromuscular dysplasia has also identified risk loci and shared genetics associated with common cardiovascular diseases such as coronary artery disease [14].

Given that fibromuscular dysplasia overwhelmingly affects women, a common belief is that there is a link between increased estrogen levels and a predisposition to the disease [8]. However, no association has currently been found between fibromuscular dysplasia and abnormalities of endogenous sex hormones or the use of oral contraceptives [8]. Pregnancy does not appear to increase this risk either [15].

Ocular manifestations of fibromuscular dysplasia are rare but can occur in the form of central or retinal artery occlusions [3]. The retinal artery, a branch of the ophthalmic artery that itself is the first branch of the intracranial part of the internal carotid artery, is the main blood supply for the inner retina and surface of the optic eye. A central retinal artery occlusion (CRAO) or branch retinal artery occlusion (BRAO) can cause painless loss of monocular vision [16]. Risk factors of CRAO and BRAO include hypertension, diabetes, and tobacco use. While the source of such occlusions is typically attributed to carotid artery atherosclerosis, cardiogenic emboli, or small artery disease, more unusual etiologies include fibromuscular dysplasia, radiation damage, or hematologic disorders. The optic nerve can also be affected by the lower blood supply, producing acute or chronic ischemic optic neuropathy. Retinal artery occlusions are generally treated with a combination of statin, antiplatelet therapy and atherosclerosis risk factor modification [5]. When retinal occlusions are associated with fibromuscular dysplasia, management may differ regarding secondary prevention measures to reduce the risk of future vascular events.

## Conclusions

Fibromuscular dysplasia is a type of arteriopathy that can present with rare but sight-threatening ocular manifestations. This case highlights the debilitating nature of these ocular manifestations, and the clinical context used to narrow the differential diagnosis. In our case, orbital MRI demonstrated flattening of the right optic nerve, while magnetic resonance angiography (MRA) head and CTA neck demonstrated a “beaded appearance" of the internal carotid artery. The patient’s presentation narrowed the differential to acute etiologies. Clinical and image study findings suggested fibromuscular dysplasia and suspected right branch retinal artery occlusion. Further research is needed to elucidate better treatment options for patients with these ocular manifestations.
